# Fostering speaking gains through flipped instruction: a mixed-methods study of self-regulation, motivation, and foreign language classroom anxiety in Chinese EFL learners

**DOI:** 10.3389/fpsyg.2026.1903309

**Published:** 2026-08-26

**Authors:** Ziyu Yuan, Yao Lu

**Affiliations:** The School of Humanities, Arts and Education, Shandong Xiehe University, Jinan, China

**Keywords:** flipped classroom, foreign language anxiety, intrinsic motivation, L2 speaking, mixed methods, positive psychology, self-regulation

## Abstract

**Introduction:**

Flipped instruction has gained traction in computer-assisted language learning, but evidence of its effects on L2 speaking and the underlying affective and motivational mechanisms remains limited, especially for young adult EFL learners in East Asia.

**Methods:**

This explanatory sequential mixed methods study examined how a 16-week flipped speaking course influenced Chinese EFL learners' speaking performance, speaking-specific self-regulation, motivation, and foreign language classroom anxiety. Seventy-four first-year English majors were assigned to a flipped group (*n* = 38) receiving pre-class video instruction and in-class speaking activities, or a control group (*n* = 36) receiving teacher-fronted instruction. Quantitative data included pretest-posttest measures of speaking, self-regulation, motivation, and foreign language classroom anxiety. Qualitative data comprised semi-structured interviews (*n* = 12) and 412 reflective journal entries, analyzed via thematic analysis with emotional valence coding.

**Results:**

Repeated measures ANOVAs showed significantly greater gains in the flipped group for speaking (ηp^2^ = 0.43), intrinsic motivation (ηp^2^ = 0.31), and self-regulation (ηp^2^ = = 0.39), but not for extrinsic motivation or anxiety. Qualitative findings identified six themes: agency in temporal control, self-monitoring via digital traces, strategic peer help seeking, enjoyment as a driver of sustained effort (vs. grade-driven behaviors), technology-mediated self-evaluation, and residual speaking anxiety-the latter three directly implicating emotional processes.

**Discussion:**

A joint display revealed convergence for self-regulation and intrinsic motivation, expansion for strategic help seeking, and discordance for extrinsic motivation. The findings suggest that flipped instruction enhances L2 speaking by fostering positive emotional engagement and reducing anxiety-driven avoidance while preserving grade-based extrinsic motivation—a pattern consistent with positive psychology perspectives on language learning. Theoretical and pedagogical implications for affective- and motivation-focused interventions in Asia–Pacific EFL contexts are discussed.

## 1 Introduction

Speaking in a second language is challenging: it demands real-time processing, fluency, and grammatical accuracy under social evaluation and public performance (Uztosun, 2020). For East Asian EFL learners, these difficulties are compounded by exam-driven systems, teacher-centered classrooms, and a collectivist culture valuing face-saving and conformity (Horwitz, 2017; Liu and Jackson, 2008; You and Dörnyei, 2016). Consequently, Chinese EFL learners often report high foreign language classroom anxiety (FLCA) and heavy reliance on extrinsic motivation, with low intrinsic interest in speaking (Liu and Li, 2024; Kang et al., 2025). Addressing these affective and motivational barriers is therefore essential for improving oral proficiency.

Flipped instruction reverses the traditional lecture-and-homework sequence: learners study pre-class videos or readings, then use class time for interactive, application-oriented activities (Bergmann and Sams, 2012; Bishop and Verleger, 2013; Strelan et al., 2020). In language teaching, this model increases comprehensible input, reduces cognitive load, and frees class time for oral practice (Chen et al., 2014; Fathi and Rahimi, 2022; Fisher et al., 2024; Turan and Akdag-Cimen, 2020). Meta-analyses confirm robust speaking gains (Vitta and Al-Hoorie, 2023), yet most evidence comes from reading, writing, or general proficiency, and few studies have examined the specific mechanisms—self-regulation, motivation, and emotion—through which flipped instruction affects L2 speaking (O'Flaherty and Phillips, 2015; Strelan et al., 2020).

Research on flipped instruction and learner emotions is thin and inconsistent. Some studies report reduced FLCA and increased motivation (Pan et al., 2022), while others warn that the self-directed pre-class phase can heighten anxiety for learners lacking self-regulatory skills (Bui and Johnson, 2024; Doman and Webb, 2017). The interplay between intrinsic and extrinsic motivation in flipped environments has received only cursory attention. Most studies treat motivation as unidimensional or focus solely on anxiety or enjoyment, rarely examining how positive and negative emotions co-evolve over time within a single intervention (Dewaele and MacIntyre, 2022; Liu, 2026). This gap is especially critical in East Asian contexts, where high-stakes exam cultures mean that extrinsic motivation often coexists with intrinsic interest rather than replacing it (Liu and Li, 2024; Kang et al., 2025). Designs that track both motivation types are clearly needed.

Furthermore, most flipped learning research originates from Western or Middle Eastern settings, leaving applicability to East Asian EFL contexts largely untested (Li et al., 2020; Pongpanich et al., 2025; You and Dörnyei, 2016). Chinese young adult EFL learners face distinct exam pressures—such as the College English Test and graduate entrance exams—alongside a growing emphasis on communicative competence (Zhang, 2019). How a flipped speaking course interacts with these pressures—whether it reduces anxiety, fosters intrinsic motivation, or adds an extra layer of responsibility—remains an open question. Moreover, most studies employ short interventions (4–8 weeks) and purely quantitative or qualitative designs, leaving knowledge of *whether* flipped instruction works outweighing understanding of *how* or *why* it works over time (Strelan et al., 2020; Vitta and Al-Hoorie, 2023). Longitudinal, mixed-methods designs combining pretest–posttest comparisons with rich qualitative data are rare but essential for uncovering the psychological processes behind flipped instruction's effects (Dörnyei, 2019; Henry and Liu, 2023).

To address these gaps, the present study employed an explanatory sequential mixed methods design to investigate the effects of a 16-week flipped speaking course on Chinese EFL learners' speaking performance, speaking specific self-regulation, intrinsic and extrinsic motivation, and foreign language classroom anxiety (FLCA). By integrating pretest posttest measures with qualitative data from reflective journals and semi structured interviews, we aimed to provide a nuanced, culturally grounded account of how flipped instruction reshapes the psychological landscape of L2 speaking in an exam driven Asian context.

## 2 Literature review

### 2.1 Theoretical frameworks: self-regulation, motivation, and emotion in L2 speaking

L2 speaking development involves interacting psychological mechanisms: self-regulation, motivation, and emotion jointly shape engagement and persistence (Dörnyei, 2005; Oxford, 2016; Pintrich, 2004). This section reviews self-regulated learning (SRL), self-determination theory (SDT), the L2 motivational self system (L2MSS), and positive psychology perspectives on emotion.

SRL refers to actively managing one's learning through cycles of planning, monitoring, control, and reflection (Pintrich, 2000, 2004; Zimmerman, 2000, 2002). Pintrich, 2004 model encompasses four phases across cognition, motivation/affect, behavior, and context, while Zimmerman (2000, 2002) specifies forethought, performance, and self-reflection. Both frameworks are context-sensitive and teachable (Panadero, 2017; Schunk and Greene, 2018). During L2 speaking, SRL tactics such as rephrasing, rehearsing, and circumlocution require mental and emotional control. SRL is vital for learner autonomy and proficiency (Dörnyei and Ryan, 2015; Teng and Zhang, 2016), yet research has focused primarily on receptive skills (Oxford, 2016; Teng and Zhang, 2022). Application of SRL to L2 speaking remains under-researched, especially outside Western settings (Teng and Zhang, 2022), though recent work links structured SRL support to improved speaking and engagement (Zhang, 2024).

SDT posits three basic needs—autonomy, competence, and relatedness—underlying self-determined behavior (Deci and Ryan, 1985; Ryan and Deci, 2000, 2017). Need satisfaction fosters intrinsic motivation and wellbeing, while frustration leads to controlled, extrinsic motivation (Ryan and Deci, 2000). SDT has been widely applied to L2 motivation (Al-Hoorie et al., 2025; Noels et al., 2003, 2019): autonomy support increases intrinsic motivation and reduces anxiety (Alamer et al., 2025; Oga-Baldwin et al., 2017), and intrinsic motivation predicts deeper processing and persistence (Noels, 2001; Noels et al., 2003, 2019). In Asian EFL contexts, autonomy-supportive classrooms are linked to greater willingness to communicate and better speaking outcomes (Mynard and Shelton-Strong, 2022). For the present study, the flipped model's self-paced pre-class videos and interactive in-class activities create autonomy and relatedness conditions that, according to SDT, should boost intrinsic motivation while leaving extrinsic motivation unchanged when external pressures (e.g., grading) remain constant (Li and Wang, 2026; Ryan and Deci, 2020).

The L2MSS shifted motivation research toward a self-based framework grounded in possible selves (Dörnyei, 2005, 2009; Higgins, 1987; Markus and Nurius, 1986). It comprises the ideal L2 self (hoped-for future speaker), the ought-to L2 self (external expectations), and the L2 learning experience (immediate, situated motives) (Dörnyei, 2009, 2019). The ideal L2 self strongly predicts motivated behavior across contexts, more so than the ought-to self (Al-Hoorie, 2018; Dörnyei and Al-Hoorie, 2017; Dörnyei and Ushioda, 2021). In Asian settings, the L2MSS has proved culturally flexible (Dörnyei and Ryan, 2015): the ideal L2 self robustly predicts motivation, while the ought-to self—shaped by parental and exam pressures—varies in its effects (Kormos and Csizér, 2014; Lamb, 2012; Lee and Hsieh, 2019; Taguchi et al., 2009; You and Dörnyei, 2016). The L2 learning experience bridges self-guides and actual behavior (Dörnyei, 2019; Kormos and Csizér, 2014; Lamb, 2012). In the flipped classroom, this experience changes markedly: pre-class video study replaces lectures, and in-class interactive tasks replace drills, potentially strengthening the ideal L2 self.

Positive psychology has foregrounded the role of emotions. Foreign language enjoyment (FLE) and foreign language classroom anxiety (FLCA) are the most studied constructs (Dewaele and MacIntyre, 2014, 2019, 2022). FLCA is situation-specific anxiety that impairs speaking, confidence, and willingness to communicate (Horwitz, 2017; Horwitz et al., 1986; Liu and Jackson, 2008; Zhang, 2019), while FLE is a positive, activating emotion that varies with instructional context (Dewaele and Dewaele, 2020; Dewaele et al., 2023). FLE and FLCA can co-occur (Boudreau et al., 2018; Dewaele and MacIntyre, 2014, 2019), and FLE is a stronger predictor of flow, participation, and speaking performance (Dewaele and MacIntyre, 2014, 2022). In Asian EFL settings, teacher support, peer collaboration, and authentic tasks boost FLE, while fear of negative evaluation raises FLCA (Jiang and Dewaele, 2019; Li, 2020; Li et al., 2020). Flow—complete absorption arising from well-matched challenge and skill—may also be facilitated by flipped classrooms (Csikszentmihalyi, 1990; Dewaele and MacIntyre, 2022). The flipped format, by offering self-paced, interactive, and collaborative learning, creates favorable conditions for both FLE and flow.

Bringing these perspectives together, the present study adopts an integrated view in which the flipped classroom simultaneously activates self-regulation, motivation, and emotion. The pre-class video phase engages SRL processes—planning, monitoring via embedded checks, and self-evaluation through voice recordings—while the autonomy to pace and repeat content and the relatedness built through interactive class tasks are expected to satisfy basic psychological needs and boost intrinsic motivation (Pintrich, 2004; Ryan and Deci, 2020; Zimmerman, 2000). Because the grading and examination system remained unchanged for both groups, SDT would predict that extrinsic motivation remains stable even as intrinsic motivation rises. The L2MSS adds a future-oriented layer: enjoyable speaking activities could make the ideal L2 self more tangible, while the ought-to self, driven by exam pressure, would continue to operate in parallel, suggesting that intrinsic and extrinsic motivation may coexist rather than trade off against each other (Dörnyei, 2019; You and Dörnyei, 2016). Positive psychology provides a lens for the emotional texture of these processes: enjoyment and pride would be expected to accompany gains in competence and autonomy, consistent with Fredrickson (2001) broaden-and-build theory, whereas anxiety may persist in high-stakes evaluative contexts, reflecting the co-occurrence of positive and negative emotions documented in prior work (Dewaele and MacIntyre, 2014). Together, SRL describes the learning processes, SDT explains the quality of motivation, the L2MSS accounts for future self-guides, and positive psychology captures the emotional underpinnings—providing a framework that guided both the design and the interpretation of the study's findings.

### 2.2 Flipped instruction for L2 speaking: affordances and empirical evidence

The flipped classroom has become common in language education (Bergmann and Sams, 2012; O'Flaherty and Phillips, 2015; Turan and Akdag-Cimen, 2020). Learners engage with pre-class videos or readings, then use class time for active tasks (Bergmann and Sams, 2012; Bishop and Verleger, 2013; Strelan et al., 2020). In language teaching, pre-class multimedia exposure is followed by in-class speaking activities (Fathi et al., 2023; Fisher et al., 2024; Turan and Akdag-Cimen, 2020). The benefits for L2 speaking are well documented: the model increases out-of-class exposure to comprehensible input, frees class time for oral production and interaction, and reduces cognitive load through self-paced video viewing (Chen et al., 2014; Fisher et al., 2024; Sweller, 1988).

Meta-analyses confirm that flipped classrooms improve language learning, with speaking gains being particularly robust (*g* = 0.58 to 1.303) (Strelan et al., 2020; Vitta and Al-Hoorie, 2023). Individual studies report positive effects on fluency, accuracy, and complexity across diverse settings, including Thailand, South Korea, and Oman (Abdullah et al., 2021; Lee and Wallace, 2018; Li and Suwanthep, 2017; Turan and Akdag-Cimen, 2020). More recent work shows that goal-oriented, interaction-embedded, and mobile-based flipped designs further enhance speaking and engagement (Rezaeyan et al., 2025; Shabani and Jabbari, 2025; Wu et al., 2017).

The pre-class phase also fosters self-regulation and autonomy by requiring time management, goal setting, and comprehension monitoring (Broadbent, 2017; Lai and Hwang, 2016). However, some students lack these skills (Bui and Johnson, 2024), and flipped designs with explicit SRL strategies or technology-enhanced systems yield stronger outcomes (Chen and Shih, 2025; Öztürk and Çakiroglu, 2021; Shyr and Chen, 2018; Tsai et al., 2025).

Regarding motivation and anxiety, findings are mixed. Some studies report increased motivation and reduced speaking anxiety (Abdullah et al., 2019, 2020, 2021; Chen and Hwang, 2020), while others find no significant anxiety reduction (Korkmaz and Mirici, 2023). Flipped grammar instruction has increased intrinsic motivation (Aldaghri, 2024), and a large Chinese quasi-experiment found improved autonomous (*d* = 1.331) and intrinsic motivation (*d* = 0.691) alongside higher test scores (Wang et al., 2025). A longitudinal study of Chinese students revealed more positive than negative emotions, mediated by teacher, peers, and technology (Qin et al., 2022). Effectiveness is further shaped by online participation, interactive behaviors, and both student-related and classroom-related factors (Lin and Hwang, 2018; Zhong, 2024, 2025).

A growing body of research in Chinese EFL settings has examined learner engagement as a multidimensional outcome. Li and Li (2022b) found that an 8-week flipped listening and speaking course improved behavioral, cognitive, and social engagement compared to a traditional class, but the gain in emotional engagement did not reach statistical significance. Li and Wang (2023) reported a similar pattern in an online flipped ESP course, with significant improvements in behavioral and cognitive engagement but not in emotional engagement, suggesting that affective engagement may be resistant to change through structural adjustments alone. Extending this line of inquiry, Li et al. (2026) integrated GenAI chatbots into a flipped speaking classroom, which led to significant gains in both emotional engagement and speaking proficiency, indicating that technology-mediated scaffolding can amplify the affective benefits of flipped instruction. These studies underscore the need for research that not only tracks engagement outcomes but also uncovers the mechanisms—both cognitive and emotional—through which flipped formats exert their effects.

Despite this evidence, gaps remain. Most studies focus on receptive skills rather than speaking-specific outcomes, and few examine speaking-specific self-regulation or emotional mechanisms (Fisher et al., 2024; O'Flaherty and Phillips, 2015; Vitta and Al-Hoorie, 2023). Moreover, a review of flipped classroom engagement research (Li and Li, 2022a) confirmed that empirical studies situated in Asian EFL settings remain scarce, and findings from Western or Middle Eastern contexts may not apply to East Asian young adult EFL learners with their exam driven cultures and teacher centered traditions (Li et al., 2020; Pongpanich et al., 2025; Zhong, 2024). An early Macau study found positive attitudes among Chinese students in flipped sections (Webb and Doman, 2016) but did not examine speaking specific self-regulation or the interplay of motivation and emotion over time. The present study addresses these gaps by investigating whether a 16 week flipped speaking course improves Chinese EFL learners' oral performance and how it shapes speaking specific self-regulation, intrinsic and extrinsic motivation, and FLCA—providing needed evidence on affective and self-regulatory mechanisms in an East Asian context.

### 2.3 Affective and motivational mechanisms in Asia–Pacific EFL contexts

The Asia–Pacific region, particularly East Asia, offers a distinctive yet underexamined setting for L2 affective and motivational research (Al-Hoorie, 2018; Dörnyei and Ushioda, 2021; Kormos and Csizér, 2014). Most research relies on Western samples and theories, raising questions about their applicability to Asian learners (Lamb, 2012; Magid, 2009; You and Dörnyei, 2016).

The Chinese EFL context has several enduring features. First, an exam-driven, high-stakes culture makes English a gatekeeper for education and jobs, so extrinsic motivation dominates (Liu and Jackson, 2008; Liu and Li, 2024; Kang et al., 2025). Second, teacher-centered, transmission-oriented pedagogy limits oral production and raises FLCA due to face concerns (Dong and Huang, 2024; Horwitz, 2017; Liu and Jackson, 2008). Third, collectivist values strengthen the ought-to L2 self (Magid, 2009; You and Dörnyei, 2016). The resulting motivational profile is distinctive: extrinsic motivation often overshadows intrinsic interest, though both coexist (Liu and Li, 2024; Kang et al., 2025). Anxiety correlates negatively with motivation, yet positive emotions exist even under pressure and mediate the link between the ideal L2 self and achievement (Chen et al., 2024; Liu, 2026; Liu and Li, 2024).

Western motivation models, including the original L2MSS, need adjustment for Asian contexts. The ought-to L2 self carries intense moral weight in China, sometimes blending with the ideal L2 self (Magid, 2009; You and Dörnyei, 2016). Researchers have proposed the dreaded L2 self to capture prevention-focused motivation (Magid, 2009), and recent work has refined the L2MSS by breaking the L2 learning experience into contextual dimensions (Li and Tsung, 2025). These refinements confirm that motivation in Asian contexts is shaped by unique cultural and educational forces, yet most flipped-learning research has not been conducted in these settings (Li et al., 2020; Pongpanich et al., 2025).

The present study adds to this context-sensitive research by testing a flipped speaking intervention in the Chinese EFL setting. By offering pre-class video access (boosting autonomy), promoting in-class interactive speaking (lowering anxiety), and encouraging self-monitoring via digital traces (supporting self-regulation), the flipped model respects the exam-driven reality while creating room for intrinsic motivation and enjoyment. Our findings—significantly larger gains in speaking, self-regulation, and intrinsic motivation—support this possibility, while the persistence of extrinsic motivation and residual anxiety confirms that contextual constraints must be understood and worked with in any effort to improve L2 speaking in Asia–Pacific settings.

### 2.4 Research gap and the present study

The literature reveals several gaps this study addresses: the target skill (speaking), psychological mechanisms (self-regulation, motivation, emotion), research designs, and populations studied. Meta-analyses confirm flipped classrooms improve language learning (Vitta and Al-Hoorie, 2023), but most evidence comes from reading, writing, or general proficiency rather than speaking (O'Flaherty and Phillips, 2015; Strelan et al., 2020; Turan and Akdag-Cimen, 2020). Speaking demands real-time processing and affective regulation in ways receptive skills do not (Uztosun, 2020), yet few studies examine whether mechanisms for grammar or vocabulary apply to oral production. Even among speaking studies, most treat self-regulation as a general trait rather than a speaking-specific process (Zhang, 2024), missing unique challenges like managing fluency and recovering from public mistakes.

Evidence on flipped instruction and learner emotions remains thin. Some reports find flipped designs reduce anxiety and boost motivation (Pan et al., 2022), while others caution that self-directed pre-class phases can increase anxiety for learners lacking self-regulatory skills (Bui and Johnson, 2024; Doman and Webb, 2017). The interplay between intrinsic and extrinsic motivation has received cursory attention; most studies treat motivation as unidimensional or focus solely on anxiety or enjoyment (Dewaele and MacIntyre, 2022; Liu, 2026). This gap is problematic for East Asian contexts, where extrinsic motivation often coexists with intrinsic interest (Kang et al., 2025). Designs tracking both motivation types are needed.

Most studies rely on short interventions (4–8 weeks) and purely quantitative or qualitative designs. Consequently, the field knows more about whether flipped instruction works than how or why over extended periods (Strelan et al., 2020; Vitta and Al-Hoorie, 2023). Longitudinal, mixed-methods designs are rare but essential for uncovering processes like strategic help seeking and the shift from grade-driven to enjoyment-driven effort (Dörnyei, 2019; Henry and Liu, 2023). Moreover, most flipped learning research comes from Western or Middle Eastern settings, leaving the model's applicability to East Asian EFL contexts largely untested (Li et al., 2020; Pongpanich et al., 2025; You and Dörnyei, 2016). Young adult EFL learners in China face distinct exam pressures and a growing emphasis on communicative competence (Zhang, 2019). How a flipped speaking course interacts with these pressures remains an open question.

To address these gaps, the present study employed an explanatory sequential mixed methods design with a 16-week intervention involving 74 first-year English majors at a Chinese university. The study was guided by two research questions:

1. Does a 16-week flipped speaking course lead to greater gains in L2 speaking performance, speaking-specific self-regulation, and intrinsic motivation, and to a greater reduction in foreign language classroom anxiety, compared to traditional teacher-fronted instruction?
2. What affective and motivational mechanisms, as revealed through learners' reflective journals and semi-structured interviews, explain the quantitative outcomes?

Quantitatively, we used a pretest posttest two group design with repeated measures ANOVAs to compare a flipped speaking group (pre-class video-based instruction plus in class activities) against a teacher fronted control group on speaking performance, speaking specific self-regulation (22 item questionnaire), intrinsic and extrinsic motivation (14 item scale), and FLCAS. Qualitatively, we collected 412 weekly reflective journals and conducted 12 semi structured interviews, analyzed via thematic analysis with emotional valence coding. A joint display integrated the findings. By examining not only whether but also how a long term flipped intervention shapes speaking outcomes, self-regulation, and the interplay of motivation and emotion in a Chinese EFL context, the study provides culturally grounded evidence. It moves beyond outcome focused comparisons to illuminate the psychological mechanisms—agency in temporal control, self-monitoring via digital traces, strategic peer help seeking, enjoyment as a driver of sustained effort, technology mediated self-evaluation, and residual speaking anxiety—that explain how flipped instruction fosters linguistic improvement and positive affective change, even within an exam driven system.

## 3 Method

An explanatory sequential mixed methods design was adopted because the study aimed first to test the effects of the flipped course on speaking, self-regulation, motivation, a nd anxiety, and then to explore the mechanisms behind those effects through learners' own accounts. The quantitative phase established whether the intervention produced significant changes, while the qualitative phase examined how and why those changes occurred—particularly the non-significant results for extrinsic motivation and anxiety (Creswell and Plano Clark, 2018). This two-phase structure is well suited to intervention research where the researcher needs both generalizable outcomes and context-sensitive explanations. The quantitative phase used a pretest–posttest, two-group experimental design to compare the effects of flipped instruction vs. teacher-fronted instruction on L2 speaking performance, self-regulation, motivation, and foreign language classroom anxiety. The qualitative phase followed, collecting semi-structured interviews and reflective journal entries to explain and elaborate the quantitative findings. Integration occurred at the interpretation stage via a joint display.

### 3.1 Participants

Participants were 74 first-year English majors recruited from a comprehensive university in eastern China (Shandong Xiehe University, Jinan, Shandong Province). All participants were native Mandarin speakers with formal English learning experience ranging from 6 to 9 years. Their English proficiency was assessed at the beginning of the semester using the Oxford Quick Placement Test (OQPT, Version 2), which classified them as intermediate (scores between 30 and 39 out of 60). None had previously experienced flipped classroom instruction in any subject, nor had they studied abroad.

Participants were randomly assigned to one of two conditions: a flipped group (*n* = 38; 32 female, 6 male; mean age = 19.1 years, SD = 0.7) or a control group (*n* = 36; 30 female, 6 male; mean age = 19.0 years, SD = 0.6). Randomization was performed using a computer-generated random number sequence (http://www.randomizer.org) by a researcher not involved in instruction. Independent-samples *t*-tests and chi-square tests confirmed that the two groups did not differ significantly at pretest on age, OQPT scores, or any dependent variable (all *p* > 0.05). All participants provided written informed consent, and the study received ethical approval from the university's Institutional Review Board. No participant withdrew during the 16-week intervention, and attendance exceeded 95% in both groups.

### 3.2 Instruments

#### 3.2.1 Speaking performance

Participants' L2 speaking performance was assessed at pretest (week 1) and posttest (week 18) using an individual oral task. Participants viewed a prompt on the LMS (e.g., a picture sequence or a short opinion question), had 2 min to prepare, and then recorded a 2-min monolog using the LMS recording tool. Two parallel versions of the prompt were created and counterbalanced across time points. Reliability between versions was established in a pilot study with 20 comparable students (Pearson's *r* = 0.86 for total scores).

Recordings were anonymised and randomized. Two trained raters—both Chinese EFL instructors blind to group assignment and time point—independently scored each recording using an analytic rubric adapted from the *IELTS Speaking Band Descriptors*. The rubric included four criteria: *Fluency and Coherence* (0–9), *Lexical Resource* (0–9), *Grammatical Range and Accuracy* (0–9), and *Pronunciation* (0–9), yielding a total score out of 36. Intraclass correlation coefficients (ICC) for the two raters were excellent: pretest ICC = 0.92 (95% CI [0.88, 0.95]), posttest ICC = 0.94 (95% CI [0.91, 0.96]). Discrepancies >2 points were resolved through discussion.

#### 3.2.2 Speaking-specific self-regulation

Self-regulation was measured using the L2 Speaking Self-Regulation Questionnaire (22 items, 6-point Likert scale from 1 = *strongly disagree* to 6 = *strongly agree*). The instrument was developed by the research team based on Pintrich, 2004 model and adapted for L2 speaking contexts. It comprises four subscales: planning (5 items, e.g., “Before I speak English, I plan what I want to say”), self-monitoring (6 items, e.g., “I pay attention to whether I am using correct grammar while speaking”), strategy use (5 items, e.g., “When I cannot find a word, I use a paraphrase or a gesture”), and self-evaluation (6 items, e.g., “After speaking, I think about what I could have done better”).

To establish initial evidence of validity, content validity was examined first. A panel of three applied linguists with expertise in self-regulated learning independently rated each item for relevance, clarity, and alignment with the intended subscale. The item-level content validity index (I-CVI) ranged from 0.86 to 1.00, and the scale-level content validity index (S-CVI/Ave) was 0.93, indicating strong expert agreement on the relevance and clarity of the items. Subsequently, an exploratory factor analysis (EFA) was performed with the pilot sample (*n* = 30) using principal axis factoring and promax rotation. The Kaiser–Meyer–Olkin measure (0.85) and Bartlett's test of sphericity (*p* < 0.001) supported the factorability of the data. Four factors with eigenvalues greater than 1.0 were extracted, corresponding to the hypothesized subscales, and together they explained 64.5% of the total variance. All items loaded on their intended factors with loadings above 0.52 and cross-loadings below 0.30.

Internal consistency was examined both in the pilot and in the main study. The pilot with 30 non-participant intermediate learners yielded a Cronbach's α of 0.89 for the total scale, with subscale αs ranging from 0.78 to 0.86. In the present study, Cronbach's α was 0.91 at pretest and 0.92 at posttest, and all subscale αs exceeded 0.78.

#### 3.2.3 Motivation

Motivation was assessed using a 14-item scale adapted from the *Intrinsic and Extrinsic Motivation in L2 Speaking Scale* (Noels et al., 2003; modified by the authors for Chinese EFL context). The scale includes two subscales: intrinsic motivation (7 items, e.g., “I speak English because I enjoy the feeling of expressing myself in another language”) and extrinsic motivation (7 items, e.g., “I speak English because I want to get a good grade in this course”). Responses were on a 6-point Likert scale. Pilot data (*n* = 35) showed good internal consistency: intrinsic α = 0.87, extrinsic α = 0.84. In the main study, pretest αs were 0.88 (intrinsic) and 0.83 (extrinsic); posttest αs were 0.90 and 0.85, respectively.

#### 3.2.4 Foreign language classroom anxiety

Anxiety was measured using the Foreign Language Classroom Anxiety Scale (FLCAS; Horwitz et al., 1986), a 33-item instrument with a 5-point Likert scale (1 = strongly disagree to 5 = strongly agree). We translated the scale into Chinese for this study; the translation was done by two bilingual applied linguists and back-translated to check accuracy, with discrepancies resolved through consensus. Sample items include “I feel very self-conscious about speaking English in front of other students” and “I get nervous and confused when I am speaking in my English class.” In the current study, Cronbach's α was 0.94 at pretest and 0.95 at posttest.

#### 3.2.5 Reflective journals

All participants in the flipped group (*n* = 38) were asked to keep a weekly reflective journal (16 weeks total). Each week, they responded to three open-ended prompts: (1) “What strategies did you use to learn the speaking micro-skill this week?” (2) “What emotions did you experience during pre-class video study and in-class speaking activities?” (3) “What helped or hindered your speaking improvement this week?” Journals were submitted via the LMS as typed entries. A total of 412 journals were collected (38 participants × 16 weeks = 608 possible; 412 = 68% compliance). Missing entries were primarily due to technical submission errors; the number of missing entries per participant ranged from 2 to 7 (M = 5.2, SD = 1.4). No participant missed more than 7 entries, and 11 participants (29%) submitted all 16 journals. Journals were anonymised by assigning participant codes (e.g., FG01–FG38) before analysis.

#### 3.2.6 Semi-structured interviews

Twelve participants from the flipped group were purposively selected for individual interviews after the posttest (week 18). Selection aimed for maximum variation: four participants with the largest gains in speaking scores (top 15%), four with moderate gains (middle 30%), and four with the smallest gains (bottom 15%). Within each stratum, equal numbers of male and female participants were selected where possible. Interviews lasted 30–45 min, were conducted in Mandarin in a quiet university meeting room, and were audio-recorded. The interview protocol included questions such as: “Can you describe a time when you felt very motivated (or anxious) during the flipped course?” “How did watching videos at home affect your willingness to speak in class?” “What role did your classmates play in your learning?” and “Did your reasons for learning English change over the 16 weeks?” Interviews were transcribed verbatim in Mandarin; relevant excerpts were translated into English for reporting.

### 3.3 Procedures

The study followed an explanatory sequential mixed-methods design. After obtaining ethical approval and written informed consent from all participants, we randomly assigned 74 first-year English majors to a flipped group (*n* = 38) or a teacher-fronted control group (*n* = 36) using a computer-generated random number sequence (http://www.randomizer.org) administered by a researcher not involved in instruction. The 16-week intervention ran during a single semester, with two 90-min sessions per week (32 sessions total). Both groups followed the same syllabus covering eight speaking micro-skills (turn-taking, hedging, discourse marking, fluency strategies, pronunciation of thought groups, narrative structuring, giving and justifying opinions, and managing repair), aligned with the College English Curriculum Requirements and topics including everyday communication, academic presentations, and cross-cultural discussion. The same instructor—a Chinese PhD candidate in Applied Linguistics with 6 years of EFL teaching experience—taught both groups to minimize teacher effects, and two research assistants observed 20% of randomly selected sessions (six per group), confirming 100% adherence to the designated instructional format. Table 1 summarizes the key differences in instructional procedures between the two groups.

**Table 1:** Comparison of instructional procedures between groups.

Aspect	Flipped group (*n* = 38)	Control group (*n* = 36)
Pre-class activities	Watch three instructor-created videos (5–7 min each) with embedded comprehension checks; submit screenshot of completion and a 1–2 min voice note practicing the micro-skill	No pre-class video materials. Homework consisted of written exercises or memorizing dialogue scripts
In-class session structure (90 min)	10 min: Instructor clarifies misconceptions from videos. 80 min: Interactive speaking activities (pair discussions, role plays, debate pyramids, fluency circles). Instructor facilitates, provides on-the-spot feedback, encourages peer interaction. No additional direct instruction unless requested	20–25 min: Lecture with PowerPoint slides. 10 min: Model examples. 15 min: Whole-class drilling or individual repetition. 40–45 min: Controlled pair work or individual workbook exercises. Instructor circulates to answer questions
Role of instructor	Facilitator; focuses on interaction and feedback	Traditional lecturer; delivers content and drills
Homework/out-of-class assignments	Pre-class videos and voice notes (required). Weekly reflective journals (qualitative data collection)	Written exercises or memorizing dialogue scripts. No journals
Qualitative data collected	Weekly reflective journals (16 weeks, 412 entries) and semi-structured interviews (*n* = 12) after posttest	None
Common features	Same syllabus (8 speaking micro-skills), same instructor, two 90-min sessions/week for 16 weeks, same pretest/posttest measures (speaking, self-regulation, motivation, anxiety)	Same as flipped group

In the flipped group, each week began with pre-class activities (approximately 45 min) delivered through the university's learning management system (ChaoXing). The instructor created three short videos (5–7 min each) explaining that week's micro-skill with models and controlled practice; each video included embedded comprehension checks (multiple-choice questions or pause-and-repeat tasks). Learners were required to submit a screenshot showing completion and a brief voice note (1–2 min) practicing the micro-skill. During the subsequent 90-min class session, the instructor spent the first 10 min clarifying common misconceptions drawn from the voice notes, and the remaining 80 min were devoted to interactive speaking activities—pair discussions, small-group role plays, debate pyramids, and fluency circles—designed to encourage spontaneous use of the pre-taught micro-skills. The instructor acted as a facilitator, providing on-the-spot feedback and encouraging peer interaction, with no additional direct instruction on the micro-skill unless requested. The control group followed a conventional teacher-fronted cycle for the same micro-skills and topics: a 20–25 min lecture with PowerPoint slides, 10 min of model examples, 15 min of whole-class drilling or individual repetition, and the final 40–45 min for controlled pair work or individual workbook exercises, with the instructor circulating to answer questions. Control participants received no pre-class video materials; their homework consisted of written exercises or memorizing dialogue scripts.

Week 1 served as the pretest phase. All participants completed the Oxford Quick Placement Test (Version 2), the speaking pretest (individual oral task with 2 min of preparation followed by a 2-min monolog, recorded via the LMS), and the three questionnaires (L2 Speaking Self-Regulation Questionnaire, Intrinsic/Extrinsic Motivation Scale, and Foreign Language Classroom Anxiety Scale) in a single 90-min computer lab session. Speaking recordings were collected individually in sound-attenuated booths. Weeks 2 through 17 comprised the intervention. Each week, the flipped group accessed their pre-class videos (due 24 h before class) and submitted weekly reflective journals through the LMS, responding to three open-ended prompts about strategies, emotions, and factors helping or hindering speaking improvement. The control group had no pre-class assignments. Both groups attended their respective in-class sessions. Instructors kept a detailed fidelity log, and the two research assistants observed sessions to ensure protocol adherence.

Week 18 was the posttest, replicating the pretest procedures exactly but with counterbalanced versions of the speaking prompt. All participants retook the speaking task and the three questionnaires under identical conditions. Weeks 19–20 were reserved for qualitative follow-up. From the flipped group, we purposively selected 12 participants for semi-structured interviews based on their speaking gain scores (four from the top 15%, four from the middle 30%, four from the bottom 15%), balancing gender where possible. Interviews lasted 30–45 min, were conducted in Mandarin in a quiet university meeting room, and were audio recorded. Meanwhile, all collected reflective journals (*n* = 412) were anonymised with participant codes. Throughout the study, attendance exceeded 95% in both groups, and no participant withdrew. All quantitative data were entered into SPSS 26.0, and qualitative data (journals and transcripts) were prepared for thematic analysis with emotional valence coding, as described in the data analysis section. Integration of the two phases occurred at the interpretation stage via a joint display.

### 3.4 Data analysis

Quantitative analyses were performed using SPSS 26.0. Preliminary checks confirmed normality (Shapiro–Wilk *p* > 0.05) and homogeneity of variances (Levene's test *p* > 0.05). Sphericity was not applicable as each repeated measures factor had only two levels (pretest, posttest). To compare changes between groups, we conducted separate 2 × 2 repeated measures ANOVAs (Time × Group) for total speaking score, fluency, self-regulation total score, intrinsic motivation, extrinsic motivation, and FLCAS total score. Partial eta squared (ηp^2^) reported effect sizes (0.01 = small, 0.06 = medium, 0.14 = large), with α =0.05 (two-tailed).

Qualitative data (412 reflective journals, 12 interview transcripts) were analyzed using Braun and Clarke (2006) six-phase thematic analysis with additional emotional valence coding. The analysis followed an iterative process. First, both researchers independently read the entire dataset to become familiar with its depth and breadth. Second, they performed line-by-line coding, generating initial codes that stayed close to the participants' language. Third, they grouped related codes into potential sub-themes and then into broader candidate themes. Fourth, they reviewed the candidate themes against the coded extracts and the full dataset to ensure they captured the data accurately. Fifth, they refined and named the final themes. Sixth, they selected vivid participant quotes and wrote the analytical narrative.

Throughout this process, emotional valence coding was applied. Any segment describing a feeling, mood, or emotional reaction was tagged with a valence label (positive, negative, or mixed) and, where possible, a discrete emotion label (e.g., enjoyment, frustration, embarrassment, pride). To make the coding process transparent, we developed a coding framework that mapped the progression from raw data to the final themes. Table 2 illustrates this framework with a selection of initial codes, sub-themes, and the resulting themes, together with emotional valence examples.

**Table 2:** Coding framework: progression from initial codes to final themes.

Initial codes (sample)	Sub-theme	Final theme	Emotional valence example
Replaying video, pausing, learning at my own speed, watching late at night	Temporal control	Agency in temporal control	Positive: “I felt relieved that I could rewind”
Checking replay count, looking at quiz accuracy, noticing weak areas	Digital trace monitoring	Self-monitoring via digital traces	Mixed: “I was ashamed at first, then motivated”
Messaging classmate on WeChat, asking partner for feedback, sharing notes	Peer help seeking	Strategic peer help seeking	Positive: “It felt safe to ask a friend”
Debates were fun, I wanted to sound natural, I forgot about grades	Enjoyment of mastery	Enjoyment as a driver of sustained effort	Positive: “I felt proud when I won the argument”
Comparing my recording with teacher's model, hearing my own improvement	Technology-mediated self-comparison	Technology-mediated self-evaluation	Mixed: “Embarrassing at first, then satisfying”
Heart racing before whole-class presentation, fear of being unprepared	Performance anxiety, Preparation anxiety	Residual speaking anxiety	Negative: “My mind went blank when the red light turned on”

The full codebook contained 47 initial codes; the table shows representative examples for each final theme.

Analyses were conducted in Mandarin; quotes were translated by a bilingual researcher and checked by a second translator. Two researchers independently read 20% of journals and all interviews, then performed line-by-line coding. Emotion-related codes were flagged for valence and labeled with discrete emotion categories. Candidate themes were generated inductively, and a valence frequency count tracked dominant emotional tone across four time blocks (weeks 1–4, 5–8, 9–12, 13–16). Themes were reviewed and refined; coder disagreements were resolved through discussion (Cohen's κ = 0.84). An audit trail documenting key analytical decisions (e.g., when codes were merged, when a theme was collapsed) was maintained throughout. Six final themes were defined with attention to emotional content. Each theme was illustrated with representative quotes and prevalence counts.

Quantitative and qualitative findings were integrated using a joint display (Creswell and Plano Clark, 2018) with three columns: quantitative results, qualitative themes, and meta-inferences.

### 3.5 Research ethics

The study was approved by the Institutional Review Board of Shandong Xiehe University, and all participants gave written informed consent. Several measures were taken to address the power imbalance inherent in the qualitative phase, given that the participants were students and the research context was a course they were taking for credit. First, the semi-structured interviews were conducted by a research assistant who was not the course instructor and who had no role in grading or evaluation. Participants were explicitly told that their interview responses and journals would not influence their course grades and that they could withdraw from the study at any time without penalty. Second, to reduce the formality of the interaction, the interviews were framed as informal conversations about the learners' experiences, and the interviewer used active listening and neutral prompting (e.g., “Can you tell me more about that?”) rather than evaluative language. Third, participant codes were used in all transcripts and journals, and any potentially identifying information was removed during transcription.

To enhance validity and reliability, we employed several strategies. Investigator triangulation was achieved by having two researchers independently code the data and then compare their coding; disagreements were resolved through discussion until consensus was reached (Cohen's κ = 0.84). Member checking was conducted with six interview participants: after preliminary themes were developed, a one-page summary in Mandarin was sent to these participants, who were invited to comment on whether the interpretations reflected their experiences; no substantive revisions were requested. The researchers also kept reflexive journals during data collection and analysis, noting their own assumptions about flipped learning and how those might influence the interpretation of the data. Finally, we provide thick description of the context, participants, and themes, supported by abundant verbatim quotes, to allow readers to assess the transferability of the findings.

## 4 Results

### 4.1 Quantitative results

Before conducting the main analyses, we tested all relevant statistical assumptions. Normality of each dependent variable at pretest and posttest was examined using the Shapiro–Wilk test separately for each group. The assumption of normality was met for all variables (all *p* > 0.05), with the sole exception of the fluency subscore in the control group at pretest (W = 0.94, *p* = 0.048). Given the robustness of repeated measures ANOVA to mild violations of normality when sample sizes exceed 30 (Tabachnick and Fidell, 2019), we did not apply any transformation. Homogeneity of variances between groups was confirmed for all pretest variables using Levene's test (all F < 2.10, all *p* > 0.05). Sphericity was not applicable because each repeated measures factor had only two levels (pretest and posttest). No univariate or multivariate outliers were identified, as all standardized residuals fell within ±2.5. We did not test for homogeneity of covariance matrices using Box's M because that assumption applies to multivariate ANOVA, not to the separate univariate repeated measures ANOVAs conducted here. Descriptive statistics for all dependent variables are presented in Table 3, and a summary of the repeated measures ANOVA results for each outcome is provided in Table 4.

**Table 3:** Descriptive statistics for speaking, self-regulation, motivation, and anxiety by group and time.

Variable	Group	Pretest M (SD)	Posttest *M* (SD)
Overall speaking (0–36)	Flipped (*n* = 38)	18.42 (2.85)	26.87 (2.31)
	Control (*n* = 36)	18.67 (2.91)	21.53 (2.64)
Fluency (0–9)	Flipped	4.21 (0.92)	7.08 (0.85)
	Control	4.34 (0.96)	5.32 (0.91)
Self-regulation total (1–6)	Flipped	3.26 (0.54)	4.58 (0.47)
	Control	3.31 (0.57)	3.79 (0.52)
Planning (1–6)	Flipped	3.15 (0.61)	4.42 (0.53)
	Control	3.19 (0.64)	3.68 (0.59)
Self-monitoring (1–6)	Flipped	3.31 (0.58)	4.67 (0.51)
	Control	3.28 (0.60)	3.85 (0.55)
Strategy use (1–6)	Flipped	3.22 (0.62)	4.51 (0.49)
	Control	3.25 (0.63)	3.72 (0.54)
Self-evaluation (1–6)	Flipped	3.35 (0.55)	4.72 (0.45)
	Control	3.39 (0.58)	3.91 (0.51)
Intrinsic motivation (1–6)	Flipped	3.05 (0.68)	4.47 (0.61)
	Control	3.10 (0.71)	3.55 (0.69)
Extrinsic motivation (1–6)	Flipped	4.32 (0.63)	4.28 (0.66)
	Control	4.28 (0.65)	4.31 (0.64)
Anxiety (FLCAS, 1–5)	Flipped	3.41 (0.59)	3.18 (0.62)
	Control	3.38 (0.61)	3.35 (0.60)

FLCAS, foreign language classroom anxiety scale. Subscale means for self-regulation are shown for completeness.

**Table 4:** Summary of repeated measures ANOVA results for each dependent variable.

Outcome	Effect	F(1,72)	*p*	ηp^2^	90% CI for ηp^2^	Observed power
Overall speaking	Time	287.43	< 0.001	0.80	[0.73, 0.85]	1.00
	Group	38.21	< 0.001	0.35	[0.22, 0.46]	1.00
	Time × Group	54.16	< 0.001	0.43	[0.30, 0.54]	1.00
Fluency	Time	162.30	< 0.001	0.69	[0.59, 0.77]	1.00
	Group	31.47	< 0.001	0.30	[0.18, 0.42]	1.00
	Time × Group	44.19	< 0.001	0.38	[0.25, 0.49]	1.00
Self-regulation (total)	Time	128.75	< 0.001	0.64	[0.53, 0.73]	1.00
	Group	27.10	< 0.001	0.27	[0.15, 0.39]	0.99
	Time × Group	46.63	< 0.001	0.39	[0.26, 0.50]	1.00
Intrinsic motivation	Time	92.11	< 0.001	0.56	[0.44, 0.66]	1.00
	Group	18.65	< 0.001	0.21	[0.09, 0.33]	0.98
	Time × Group	32.45	< 0.001	0.31	[0.18, 0.43]	1.00
Extrinsic motivation	Time	0.08	0.780	0.001	[0.00, 0.03]	0.06
	Group	0.05	0.823	< 0.001	[0.00, 0.02]	0.06
	Time × Group	0.36	0.552	0.005	[0.00, 0.04]	0.09
Anxiety (FLCAS)	Time	7.84	0.007	0.10	[0.02, 0.21]	0.79
	Group	0.45	0.504	0.006	[0.00, 0.06]	0.10
	Time × Group	2.41	0.125	0.032	[0.00, 0.11]	0.34

Confidence intervals for ηp^2^ are reported as 90% CI following recommended practice. Observed power is based on α = 0.05.

#### 4.1.1 Speaking performance

We conducted a 2 (Time: pretest, posttest) × 2 (Group: flipped, control) repeated measures ANOVA on the total speaking score (maximum 36). There was a significant main effect of time, F(1,72) = 287.43, *p* < 0.001, ηp^2^ = 0.80, 90% CI [0.73, 0.85], indicating that both groups improved from pretest to posttest. The main effect of group was also significant, F(1,72) = 38.21, *p* < 0.001, ηp^2^ = 0.35, 90% CI [0.22, 0.46], with the flipped group scoring higher on average across both time points. Critically, the Time × Group interaction was large and significant, F(1,72) = 54.16, *p* < 0.001, ηp^2^ = 0.43, 90% CI [0.30, 0.54], confirming that the flipped group made significantly greater gains than the control group. Simple effects analysis using a Bonferroni-adjusted alpha of 0.025 revealed that the flipped group improved from a pretest mean of 18.42 (SD = 2.85) to a posttest mean of 26.87 (SD = 2.31), mean difference = 8.45, 95% CI [7.21, 9.69], t(37) = 13.58, *p* < 0.001, Cohen's *d* = 3.18. The control group also improved, from 18.67 (SD = 2.91) to 21.53 (SD = 2.64), mean difference = 2.86, 95% CI [1.94, 3.78], t(35) = 6.32, *p* < 0.001, Cohen's *d* = 1.02. At posttest, the flipped group significantly outperformed the control group, mean difference = 5.34, 95% CI [4.12, 6.56], t(72) = 8.76, *p* < 0.001, Cohen's *d* = 2.08.

Given that fluency is a theoretically central component of oral production and a primary target of the flipped intervention, we performed a separate ANOVA on the fluency subscore (0–9). The Time × Group interaction was again large and significant, F(1,72) = 44.19, *p* < 0.001, ηp^2^ = 0.38, 90% CI [0.25, 0.49]. Simple effects indicated that the flipped group's fluency increased from 4.21 (SD = 0.92) to 7.08 (SD = 0.85), mean difference = 2.87, 95% CI [2.45, 3.29], t(37) = 13.92, *p* < 0.001, Cohen's *d* = 3.27. The control group's fluency increased from 4.34 (SD = 0.96) to 5.32 (SD = 0.91), mean difference = 0.98, 95% CI [0.62, 1.34], t(35) = 5.50, *p* < 0.001, Cohen's *d* = 1.05. At posttest, the flipped group outperformed the control group by 1.76 points, 95% CI [1.29, 2.23], t(72) = 7.44, *p* < 0.001, Cohen's *d* = 1.98.

#### 4.1.2 Speaking-specific self-regulation

The same ANOVA design applied to the total self-regulation score (1–6) yielded a significant Time × Group interaction, F(1,72) = 46.63, *p* < 0.001, ηp^2^ = 0.39, 90% CI [0.26, 0.50]. The flipped group's total self-regulation increased from 3.26 (SD = 0.54) to 4.58 (SD = 0.47), mean difference = 1.32, 95% CI [1.09, 1.55], t(37) = 11.48, *p* < 0.001, Cohen's *d* = 2.64. The control group also improved, from 3.31 (SD = 0.57) to 3.79 (SD = 0.52), mean difference = 0.48, 95% CI [0.28, 0.68], t(35) = 4.82, *p* < 0.001, Cohen's *d* = 0.87. At posttest, the flipped group scored significantly higher than the control group, mean difference = 0.79, 95% CI [0.51, 1.07], t(72) = 5.57, *p* < 0.001, Cohen's *d* = 1.38. To examine whether the intervention affected specific self-regulation subcomponents, we conducted follow-up ANOVAs on the four subscales (planning, self-monitoring, strategy use, self-evaluation). All four showed significant Time × Group interactions: planning, F(1,72) = 28.14, *p* < 0.001, ηp^2^ = 0.28; self-monitoring, F(1,72) = 41.73, *p* < 0.001, ηp^2^ = 0.37; strategy use, F(1,72) = 32.56, *p* < 0.001, ηp^2^ = 0.31; self-evaluation, F(1,72) = 38.92, *p* < 0.001, ηp^2^ = 0.35. The flipped group's gains were largest for self-evaluation (mean increase = 1.37, SD = 0.48) and self-monitoring (mean increase = 1.36, SD = 0.52), whereas the control group's gains were modest across all subscales (mean increases ranging from 0.45 to 0.52).

#### 4.1.3 Motivation

For intrinsic motivation, the Time × Group interaction was significant and large, F(1,72) = 32.45, *p* < 0.001, ηp^2^ = 0.31, 90% CI [0.18, 0.43]. The flipped group's intrinsic motivation rose sharply from 3.05 (SD = 0.68) to 4.47 (SD = 0.61), mean difference = 1.42, 95% CI [1.15, 1.69], t(37) = 10.53, *p* < 0.001, Cohen's *d* = 2.22. The control group showed a more modest increase, from 3.10 (SD = 0.71) to 3.55 (SD = 0.69), mean difference = 0.45, 95% CI [0.21, 0.69], t(35) = 3.79, *p* < 0.001, Cohen's *d* = 0.65. At posttest, the flipped group reported significantly higher intrinsic motivation than the control group, mean difference = 0.92, 95% CI [0.59, 1.25], t(72) = 5.58, *p* < 0.001, Cohen's *d* = 1.35. In contrast, for extrinsic motivation, the Time × Group interaction was not significant, F(1,72) = 0.36, *p* = 0.552, ηp^2^ = 0.005, 90% CI [0.00, 0.04]. Both groups remained stable: flipped group pretest M = 4.32 (SD = 0.63) to posttest M = 4.28 (SD = 0.66); control group pretest M = 4.28 (SD = 0.65) to posttest M = 4.31 (SD = 0.64). The main effects of time (F(1,72) = 0.08, *p* = 0.780, ηp^2^ = 0.001) and group (F(1,72) = 0.05, *p* = 0.823, ηp^2^ < 0.001) were also non-significant. Thus, the flipped format did not alter extrinsic motivation differently from traditional instruction.

#### 4.1.4 Foreign language classroom anxiety

The ANOVA on FLCAS total scores (1–5) revealed a significant main effect of time, F(1,72) = 7.84, *p* = 0.007, ηp^2^ = 0.10, 90% CI [0.02, 0.21], indicating a small overall decrease in anxiety. The main effect of group was not significant, F(1,72) = 0.45, *p* = 0.504, ηp^2^ = 0.006. Critically, the Time × Group interaction was not significant, F(1,72) = 2.41, *p* = 0.125, ηp^2^ = 0.032, 90% CI [0.00, 0.11]. Although the flipped group showed a numerically larger reduction (pretest M = 3.41, SD = 0.59; posttest M = 3.18, SD = 0.62) compared to the control group (pretest M = 3.38, SD = 0.61; posttest M = 3.35, SD = 0.60), the difference in change did not reach statistical significance. The observed power for the interaction was 0.34, suggesting that the study was underpowered to detect a small effect. To explore whether specific anxiety subtypes changed, we examined the three commonly reported FLCAS subscales (communication apprehension, test anxiety, fear of negative evaluation). Only fear of negative evaluation showed a near-significant group-by-time trend, F(1,72) = 3.82, *p* = 0.055, ηp^2^ = 0.05, favoring the flipped group. However, given the overall non-significant findings, we conclude that the flipped course did not significantly reduce foreign language classroom anxiety more than traditional instruction.

Overall, the repeated measures ANOVAs demonstrated that the flipped group outperformed the control group in overall speaking (ηp^2^ = 0.43), fluency (ηp^2^ = 0.38), self-regulation (ηp^2^ = 0.39), and intrinsic motivation (ηp^2^ = 0.31). All these effects were statistically significant and practically meaningful. In contrast, no significant group-by-time interaction emerged for extrinsic motivation or foreign language classroom anxiety, suggesting that the flipped format did not alter these constructs differently from teacher-fronted instruction. These quantitative results provide the foundation for the qualitative phase, which aims to explain the mechanisms behind the observed gains and the unexpected stability of extrinsic motivation and anxiety.

### 4.2 Qualitative results

The qualitative dataset comprised 412 reflective journal entries (collected weekly from 38 flipped group participants) and 12 semi-structured interviews conducted at posttest. Thematic analysis, augmented with emotional valence coding, yielded six themes that explain the quantitative patterns: (1) agency in temporal control, (2) self-monitoring via digital traces, (3) strategic peer help-seeking, (4) enjoyment as a driver of sustained effort (contrasted with grade-driven behaviors), (5) technology-mediated self-evaluation, and (6) residual speaking anxiety. Below, each theme is described with representative quotes, participant codes (FG = flipped group, followed by participant number; journal entries are denoted by week number), and, where relevant, prevalence counts and emotional valence trajectories.

Emotional valence coding of the 412 journal entries revealed a distinct shift over the 16 weeks. In weeks 1–4, 43% of entries were coded as predominantly negative (e.g., frustration, nervousness, self-doubt), 32% as mixed, and 25% as positive. By weeks 13–16, positive entries rose to 61%, mixed entries accounted for 28%, and negative entries dropped to 11%. This trajectory aligns with the quantitative increase in intrinsic motivation and self-regulation, while the persistence of negative valence in a minority of entries foreshadows the theme of residual anxiety.

#### 4.2.1 Theme 1: agency in temporal control

Participants consistently described the pre-class video format as affording them a sense of control over *when* and *how long* they engaged with input—a sharp contrast to the fixed pace of teacher-fronted lectures. This agency was not merely about convenience; it enabled deliberate rehearsal and reduced the pressure of real-time comprehension.

“In the traditional classroom, the teacher explains a grammar point once, and if I blink, I miss it. With the videos, I can watch at 10 pm after my other homework, or early in the morning. I can pause after every sentence and repeat the phrase until my mouth gets used to it.” (FG07, interview)

“Week 3: I replayed the part about thought groups three times. The first time I did not understand. The second time I imitated. The third time I recorded myself and compared. I would never dare to ask the teacher to repeat a live explanation three times—she would think I am stupid.” (FG15, journal, week 3)

This agency was mentioned by all 12 interviewees and appeared in 78% of journals (at least once per participant). Crucially, participants linked this control to reduced speaking anxiety and increased willingness to attempt challenging structures—a mechanism that quantitatively manifested as greater fluency gains (ηp^2^ = 0.38). Emotional valence coding showed that entries mentioning temporal agency were 3.2 times more likely to be coded as positive than negative.

#### 4.2.2 Theme 2: self-monitoring via digital traces

Unlike traditional homework, the video platform generated visible traces of their own behavior (pause counts, replay frequency, quiz scores), which participants repurposed as self-monitoring tools. This theme explains the large gain in self-regulation (ηp^2^ = 0.39), particularly the self-monitoring subscale.

“I noticed that every time the video asked a question about hedging, I paused. The platform showed me a red dot for low accuracy. That made me realize: I am weak at softening my opinions. So before the next class, I watched that segment again and wrote down three hedging phrases.” (FG22, interview)

“Week 7: My replay count for the pronunciation video was 11 times. I felt ashamed at first, but then I thought—this data is honest. I cannot cheat myself. So I forced myself to practice until my replay count went down.” (FG31, journal, week 7)

Participants used these digital traces not as external evaluations but as *objective mirrors* of their own learning process. This contrasts with teacher-fronted settings where self-assessment often relies on vague feelings (“I think I understood”). Eight of the 12 interviewees explicitly stated that seeing their own interaction data changed how they studied. Emotional valence shifted over time: initial encounters with low quiz scores produced frustration (negative valence), but by weeks 8–10, participants described using the same data as motivation (“I saw my improvement—replay count dropped from 11 to 3—that felt great”).

#### 4.2.3 Theme 3: strategic peer help-seeking

While the flipped group was not explicitly designed to promote collaboration beyond in-class activities, participants spontaneously developed a peer help-seeking strategy that was absent in the control condition. Unlike traditional help-seeking (asking the teacher), this was strategic, low-stakes, and often technology-mediated.

“I did not understand the difference between ‘will' and ‘be going to' from the video. Instead of waiting for class, I messaged two classmates on WeChat. One sent me a screenshot of her notes. The other shared a short video she found on Bilibili. In the old class, I would just keep quiet.” (FG12, interview)

“Week 10: Before the debate activity, I asked my partner to listen to my argument and tell me if my intonation sounded questioning or confident. She recorded a voice message for me. That is faster than waiting for the teacher.” (FG05, journal, week 10)

This theme appeared in 9 of 12 interviews and 62% of journals. Importantly, help-seeking was not a sign of dependency but a strategic move to resolve gaps *before* class, which in turn increased their confidence during in-class speaking. Quantitatively, this behavior may explain the expansion effect noted in the joint display: peer help-seeking was not captured by the self-regulation questionnaire (which focused on individual strategies), yet it substantially contributed to speaking gains.

#### 4.2.4 Theme 4: enjoyment as a driver of sustained effort (vs. grade-driven behaviors)

This theme directly explains the large increase in intrinsic motivation (ηp^2^ = 0.31) and the discordance with extrinsic motivation. Participants repeatedly distinguished between studying *for a grade* (extrinsic) and studying *because the process became enjoyable* (intrinsic). The flipped format, through its combination of self-paced video and interactive class activities, shifted their motivational orientation over time.

“At the beginning, I watched the videos only because the teacher would check our completion. I wanted the participation points. But around week 5, I started to actually look forward to the in-class speaking tasks. The debate pyramid was so fun—I forgot I was ‘learning English'. I just wanted to win the argument.” (FG18, interview)

“Week 12: Today I recorded my voice note three times because I wanted it to sound natural, not because the teacher asked. I never did that before. I used to just submit the first take.” (FG09, journal, week 12)

“Grades are still important—I need a high GPA to apply for graduate school. But now, when I practice speaking, I am not thinking about the exam. I am thinking about how to tell a story smoothly. That feeling is new.” (FG27, interview)

All 12 interviewees reported a shift from “grade pressure” to “enjoyment of mastery” at some point, though the timing varied (weeks 5–9 for most). Emotional valence coding confirmed that journal entries containing enjoyment markers (e.g., “fun”, “interesting”, “satisfying”, “proud”) increased from 18% in weeks 1–4 to 67% in weeks 13–16. Importantly, participants did not abandon extrinsic goals—their extrinsic motivation scores remained stable (quantitative non-significant interaction)—but they added a new, more sustainable driver. This explains the discordance: the flipped course built intrinsic motivation on top of pre-existing extrinsic orientation, rather than replacing one with the other.

#### 4.2.5 Theme 5: technology-mediated self-evaluation

Beyond self-monitoring (Theme 2), participants engaged in deliberate self-evaluation using the technological affordances of the LMS. Unlike traditional self-evaluation (e.g., “I think I did well”), the video and voice recording tools allowed them to compare their own performance against models and against their own past performances.

“The voice note feature was the most useful. After I recorded, I could listen to myself—my voice, my pauses, my wrong sounds. At first I hated hearing my own voice. But then I started comparing week 1 recording with week 8. The difference was shocking. I could hear that I was faster and clearer.” (FG03, interview)

“Week 14: I recorded my answer to the opinion question, then played the teacher's model answer. I noticed that my sentences were too short. So I rewrote my answer, recorded again, and this time I added connectors like ‘however' and ‘moreover'. The platform let me do this side by side.” (FG34, journal, week 14)

This theme was present in 10 of 12 interviews and in 71% of journals. It directly supports the quantitative self-regulation gain, specifically the self-evaluation subscale. Emotionally, initial encounters with one's own recorded voice produced embarrassment or even shame (negative valence: “I sounded like a robot”). However, by mid-intervention, participants described a shift to pride and satisfaction as they detected improvement. This emotional shift—from negative to positive valence in self-evaluation—aligns with the broader trajectory of increasing intrinsic motivation.

#### 4.2.6 Theme 6: residual speaking anxiety

Despite significant gains in speaking performance and intrinsic motivation, and a numerical but non-significant reduction in FLCAS scores (ηp^2^ = 0.032), anxiety did not disappear. This theme explains why the quantitative interaction for anxiety was non-significant: the flipped format reduced some sources of anxiety but introduced or left others intact.

Participants described two distinct types of residual anxiety:

**Type A—Performance anxiety in high**-**stakes situations:** Even after 16 weeks, speaking in front of the whole class or being recorded for formal assessment triggered nervousness.

“I am much more confident in pair work and small groups. But when the teacher says ‘now each of you will present to the class', my heart still races. The flipped course did not cure that. Maybe nothing can.” (FG19, interview)

“Week 15: The mock final speaking test was recorded. I knew I had improved—my partner told me—but as soon as the red recording light turned on, my mind went blank for five seconds. I hate that feeling.” (FG11, journal, week 15)

**Type B—Anxiety about being under**-**prepared:** Some participants reported that the flipped format placed the onus of preparation on them, which created a new kind of worry: fear of coming to class unprepared and being exposed.

“In the traditional class, I could just sit and listen passively. Now, if I do not watch the video, I will be lost during the in-class activities. Sometimes I felt anxious about whether I had understood the video correctly. What if I practiced the wrong thing?” (FG25, interview)

“Week 6: I was sick and could not watch the video. I went to class anyway and felt terrible. Everyone else was doing the role play fluently. I just nodded. That anxiety stayed with me for 2 days.” (FG30, journal, week 6)

Despite these residual anxieties, participants consistently reported that the *overall* experience was less anxiety-provoking than traditional instruction (as reflected in the significant main effect of time, F(1,72) = 7.84, *p* = 0.007, ηp^2^ = 0.10). However, because the control group also experienced a small anxiety reduction (possibly due to familiarity with the instructor or natural habituation), the interaction did not reach significance. The qualitative data clarify that the flipped format *re*-*distributes* anxiety rather than eliminating it: it reduces lecture-induced boredom and fear of being called on unprepared, but introduces anticipatory anxiety about pre-class mastery and persistent performance pressure in formal evaluation contexts.

Taken together, the six themes explain the quantitative patterns. Agency and self-monitoring via digital traces account for gains in self-regulation (ηp^2^ = 0.39) and fluency (ηp^2^ = 0.38). Strategic peer help seeking offers an unmeasured pathway to improvement (expansion meta-inference). Enjoyment as a driver of sustained effort explains the rise in intrinsic motivation (ηp^2^ = 0.31) and, together with stable extrinsic motivation, the discordance between quantitative and qualitative findings. Technology-mediated self-evaluation reinforces the self-regulation result and shows an emotional shift from embarrassment to pride, consistent with positive psychology. Residual speaking anxiety clarifies why anxiety reduction did not differ significantly between groups: the flipped format reduces some anxieties but introduces or preserves performance pressure and preparation worry.

## 5 Discussion

This mixed methods study examined how a 16-week flipped speaking course affected Chinese EFL learners' speaking, self-regulation, intrinsic/extrinsic motivation, and anxiety. Quantitatively, the flipped group outperformed the control group in speaking (ηp^2^ = 0.43), fluency (ηp^2^ = 0.38), self-regulation (ηp^2^ = 0.39), and intrinsic motivation (ηp^2^ = 0.31), but not in extrinsic motivation or anxiety. Qualitatively, six themes—agency in temporal control, self-monitoring via digital traces, strategic peer help seeking, enjoyment as a driver of sustained effort, technology-mediated self-evaluation, and residual speaking anxiety—explain these patterns. The large effects for speaking and fluency align with meta-analyses showing flipped classrooms produce robust speaking gains (Strelan et al., 2020; Vitta and Al-Hoorie, 2023). Our effect sizes exceed those of many previous studies (e.g., Abdullah et al., 2019; Li and Suwanthep, 2017), likely due to the 16-week duration and focus on speaking-specific micro skills. The theme of agency in temporal control—learners valued pausing and replaying videos—reduced real-time pressure and enabled deliberate rehearsal, consistent with cognitive load theory (Chen et al., 2014; Sweller, 1988). Unlike fixed-pace lectures, the flipped format allowed lower-proficiency learners to revisit explanations without social embarrassment (FG15). Thus, fluency gains (ηp^2^ = 0.38) stem from repeated, low-stakes video practice.

Self-monitoring via digital traces (replay counts, quiz scores) transformed platform data into self-regulatory feedback, supporting Zimmerman's (2000, 2002) claim that self-observation is core to SRL. Objective digital traces made weaknesses visible, contributing to the large gain in speaking-specific self-regulation (ηp^2^ = 0.39), especially in self-monitoring and self-evaluation. This matches studies with explicit SRL support (Chen and Shih, 2025; Öztürk and Çakiroglu, 2021; Tsai et al., 2025). Unlike those studies, our intervention provided no explicit SRL training; technology affordances alone prompted spontaneous self-regulation, extending prior work (Broadbent, 2017; Lai and Hwang, 2016).

Strategic peer help seeking emerged as an unmeasured pathway. Participants used WeChat to resolve pre-class misunderstandings—a proactive, low-stakes strategy absent in the control group. Prior research noted flipped classrooms foster collaboration (Shabani and Jabbari, 2025; Wu et al., 2017), but our study specifies that peer help seeking becomes strategic when learners face content gaps. This is especially important in collectivist Asian contexts where direct teacher questioning may be avoided due to face concerns (Horwitz, 2017; Liu and Jackson, 2008). Thus, the flipped model indirectly supports relatedness—a core SDT need—through peer networks. Intrinsic motivation rose sharply (ηp^2^ = 0.31) while extrinsic motivation remained stable. This pattern—intrinsic gain without extrinsic decline—challenges the assumption that the two are antagonistic (Deci et al., 2001; Ryan and Deci, 2017). In exam-driven Asian contexts, extrinsic motivation (grades) serves as a persistent baseline, and the flipped course added an enjoyment layer. The flipped format did not modify the assessment criteria or the high-stakes examination system that surrounds Chinese higher education; consequently, the external pressures that sustain extrinsic motivation were unchanged. The theme enjoyment as a driver of sustained effort illustrates this shift: initially students watched videos for points, but by weeks 5–9 they looked forward to debates. FG18's comment (“I forgot I was ‘learning English'. I just wanted to win the argument”) captures flow (Csikszentmihalyi, 1990; Dewaele and MacIntyre, 2022). From SDT, the flipped format satisfied autonomy (self-paced viewing), competence (low-risk practice, immediate feedback), and relatedness (interactive activities, peer help). These need satisfactions increase intrinsic motivation (Alamer et al., 2025; Noels et al., 2019; Ryan and Deci, 2020). However, SDT also holds that satisfying basic needs enhances autonomous motivation but does not necessarily reduce controlled motivation unless the external controlling forces are explicitly removed (Ryan and Deci, 2020). Since the grading structure and examination pressures were identical for both groups, extrinsic motivation remained a powerful driver alongside the emerging intrinsic interest. The persistence of extrinsic motivation aligns with studies of Chinese EFL learners showing coexistence of both types (Liu and Li, 2024; Kang et al., 2025). Qualitative data confirmed this dual orientation: participants such as FG27 stated that “grades are still important—I need a high GPA to apply for graduate school,” yet they also described a newfound enjoyment in speaking practice. Thus, researchers and practitioners in East Asia should view intrinsic and extrinsic motivation as parallel drivers, not mutually exclusive. The discordance between the non-significant quantitative interaction and qualitative shift for extrinsic motivation is resolved by qualitative data: grades remained important, but reasons for practice expanded—a nuance the self-regulation questionnaire missed, highlighting the strength of mixed methods integration (Creswell and Plano Clark, 2018).

The non-significant interaction for FLCA (ηp^2^ = 0.032, *p* = 0.125)—despite a larger numerical reduction in the flipped group (3.41 → 3.18) than control (3.38 → 3.35)—is explained by residual speaking anxiety. Participants reported two persisting anxiety types: performance anxiety in high-stakes situations (e.g., whole-class presentations) and anxiety about being under-prepared (fear of coming to class without watching the video). The first type is common (Horwitz, 2017; Zhang, 2019) and may not be fully alleviated in 16 weeks. The second type is a unique flipped-model demand; similar concerns appear in Bui and Johnson (2024) and Doman and Webb (2017). The control group also showed a small anxiety reduction (main effect of time, ηp^2^ = 0.10), possibly due to habituation. Thus, the flipped format did not differentially reduce anxiety, even though qualitative data suggested some sources (e.g., fear of being called on unprepared) diminished. A near-significant trend for fear of negative evaluation (*p* = 0.055) hints that the flipped format reduced social judgment concerns, a key FLCA source in collectivist cultures (Dong and Huang, 2024; Jiang and Dewaele, 2019). Future research should use more context-sensitive anxiety measures. Technology-mediated self-evaluation—using voice recordings to compare current performance with past or model answers—supported the large gain in the self-evaluation subscale (ηp^2^ = 0.35). Emotionally, initial embarrassment shifted to pride as learners detected improvement, aligning with Fredrickson's broaden-and-build theory (2001). Our emotional valence coding over 16 weeks showed negative entries dropping from 43% (weeks 1–4) to 11% (weeks 13–16) and positive entries rising from 25% to 61%. This longitudinal pattern is rarely documented in flipped learning research, which typically uses post-only surveys (e.g., Abdullah et al., 2019; Wang et al., 2025). The observed emotion-cognition dynamics echo Qin et al., 2022, where positive emotions promoted cognitive functions and negative emotions did not always lead to disengagement due to sociocultural mediators (teacher responsiveness, peer support, technology).

Our findings challenge the stereotype that Asian EFL learners are passively reliant on teacher-fronted instruction. The flipped group thrived in an environment demanding self-regulation and active participation, countering claims that flipped models are culturally inappropriate for East Asia (Li et al., 2020; Pongpanich et al., 2025). However, persistent residual anxiety and stable extrinsic motivation show that contextual constraints—exam pressure, face concerns—do not vanish. The anxiety about being under-prepared reflects increased student responsibility, which may feel heavier in cultures with strong conformity and external expectations (Magid, 2009; You and Dörnyei, 2016). Thus, flipped instruction in China requires scaffolding for struggling students and mitigation of new anxieties. The L2MSS offers a lens: the ideal L2 self likely gained salience through enjoyable activities, while the ought-to L2 self (exam performance) remained stable, explaining stable extrinsic motivation. The transformed L2 learning experience may have strengthened the ideal L2 self over time (Dörnyei, 2019; Henry and Liu, 2023). Our qualitative data show learners beginning to imagine themselves as fluent speakers (“I am thinking about how to tell a story smoothly”), suggesting a shift in self-concept. Future L2MSS research in Asia should incorporate measures of the L2 learning experience and track how instructional innovations reshape self-guides.

## 6 Limitations and future research

Several limitations must be acknowledged. First, the study was conducted at a single Chinese university with intermediate English majors, limiting generalizability to other proficiency levels, disciplines, or institutions. Replication with lower proficiency learners, non-English majors, and across different Asian countries (e.g., Japan, Vietnam, Indonesia) is needed. Second, the intervention lasted 16 weeks—longer than many flipped studies—but still could not capture long-term retention of speaking gains or sustained motivational changes beyond one semester. Longitudinal follow-up (e.g., 6 or 12 months post-intervention) would clarify whether the intrinsic motivation boost decays or persists. Third, the FLCAS may not be sensitive enough to detect reductions in low-stakes speaking anxiety; future research should use context-specific anxiety measures (e.g., anxiety during video recording vs. whole-class presentation) and physiological indicators (e.g., heart rate, skin conductance) to triangulate findings. Fourth, the observed power for the anxiety interaction was low (0.34), indicating that the study was underpowered to detect a small effect. A larger sample or a more sensitive measure might reveal a significant reduction. Fifth, the self-regulation questionnaire was developed by the research team. Although content validity was supported by expert review and an exploratory factor analysis with the pilot sample (*n* = 30) yielded a clear four-factor structure, the small sample precluded a confirmatory factor analysis. Future studies with a larger independent sample (ideally *N* ≥ 200) should subject the instrument to CFA and further psychometric testing to establish its construct validity more firmly. Sixth, the reflective journal compliance rate (68%) was modest, though the missing data were randomly distributed and no participant missed more than seven entries. Future studies should use automated reminders or in-class journaling to increase compliance.

Additionally, the role of the instructor—a Chinese PhD candidate with 6 years of experience—may have influenced results. Although the same instructor taught both groups to control for teacher effects, the instructor's enthusiasm for the flipped method could have introduced subtle bias. Replication with multiple instructors and blinded conditions is desirable. Finally, the study did not measure flow or foreign language enjoyment directly, relying on qualitative inferences. Future mixed-methods studies should include validated scales for FLE and flow to quantify the emotional mechanisms (Dewaele and MacIntyre, 2014, 2022; Li, 2020).

## 7 Pedagogical implications

The findings of this study carry several practice-oriented recommendations, each grounded directly in the quantitative outcomes or the qualitative themes. First, because the qualitative theme of agency in temporal control showed that learners valued the ability to pause, rewind, and rehearse at their own pace, pre-class videos should be kept short (5–7 min) and include embedded comprehension checks. This design, which was used in the present study and directly linked to gains in fluency (ηp^2^ = 0.38), allows learners to manage their own learning rhythm and reduces the anxiety associated with real-time lecture comprehension.

Second, the theme of self-monitoring via digital traces revealed that learners spontaneously used platform data—such as replay counts and quiz accuracy—to identify weak areas and guide further practice. This behavior contributed to the large gain in self-regulation (ηp^2^ = 0.39). Instructors should therefore explicitly teach students how to interpret these digital traces as self-regulatory tools, rather than assuming students will discover this on their own. A brief in-class orientation to the analytics dashboard and periodic reflective prompts (e.g., “What does your replay data tell you about your learning this week?”) could amplify this natural tendency. Third, the sharp increase in intrinsic motivation (ηp^2^ = 0.31) and the qualitative shift from grade-driven to enjoyment-driven effort indicate that in-class activities should be interactive and genuinely enjoyable. Formats such as debate pyramids, fluency circles, and role-plays generated positive emotions that fuelled intrinsic motivation. The present study therefore echoes the call for flipped classrooms to replace passive drills with communication-focused, game-like tasks that create the conditions for flow (Csikszentmihalyi, 1990).

Fourth, the persistent residual anxiety—both performance anxiety in formal assessment situations and anxiety about being under-prepared—was a clear finding from the qualitative phase. To mitigate this, instructors should create frequent low-stakes speaking opportunities (e.g., ungraded pair work, voice note submissions not shared publicly) so that learners build confidence before facing high-stakes evaluations. Additionally, acknowledging the anxiety about under-preparation by allowing occasional flexible deadlines or built-in “catch-up” weeks can reduce the fear of being left behind without lowering the overall expectation for pre-class engagement.

Fifth, the theme of strategic peer help seeking, which emerged as an unmeasured mechanism contributing to speaking gains, suggests that collaborative learning should be actively encouraged. Instructors can set up structured WeChat groups or online discussion forums where learners can ask questions, share notes, and give peer feedback before class. This approach leverages the collectivist orientation of Chinese learners and turns it into a pedagogical asset rather than an obstacle to active participation. Finally, the stable extrinsic motivation indicates that the flipped course did not weaken the drive for grades. A realistic pedagogical goal in exam-driven East Asian contexts is therefore to layer intrinsic motivation on top of existing extrinsic goals. Practitioners should design assessments that continue to reward achievement while also carving out space for enjoyment-based speaking tasks that are not formally graded. This dual-motivation model is more attainable than attempting to replace extrinsic drivers entirely.

## 8 Conclusions

This explanatory sequential mixed methods study provides robust evidence that a 16-week flipped speaking course significantly improves Chinese EFL learners' speaking performance, fluency, speaking-specific self-regulation, and intrinsic motivation, while leaving extrinsic motivation stable. The qualitative themes—agency in temporal control, self-monitoring via digital traces, strategic peer help seeking, enjoyment as a driver of sustained effort, technology-mediated self-evaluation, and residual speaking anxiety—elucidate the psychological mechanisms underlying these effects. The non-significant change in extrinsic motivation, shown to coexist with a substantial rise in intrinsic motivation, suggests a dual-motivation profile that is well-suited to exam-oriented educational systems. The non-significant reduction in anxiety, explained by residual performance pressure and preparation worry, underscores that flipped instruction is not a panacea but a pedagogical shift that must be accompanied by emotional scaffolding. Together, these findings show that a carefully structured flipped speaking course can foster both linguistic gains and positive self-regulatory and motivational change without requiring a dismantling of existing assessment structures. Future research should extend these findings to diverse Asian contexts, explore longer-term trajectories, and develop targeted interventions for residual anxiety. For practitioners, the study offers a set of evidence-aligned strategies—short self-paced videos with embedded checks, explicit digital trace training, enjoyment-focused interactive tasks, low-stakes speaking practice, structured peer collaboration, and dual-motivation assessment—that can be adopted within current curricular constraints.

## Data Availability

The data analyzed in this study is subject to the following licenses/restrictions: The quantitative datasets generated and analyzed during the current study (including speaking scores, questionnaire responses, and ANOVA outputs) are available from the corresponding author upon reasonable request. The qualitative data (reflective journals and interview transcripts) are not publicly available because they contain sensitive personal reflections, even after anonymisation, and participants did not agree to public archiving. However, anonymised excerpts are provided in the manuscript as illustrative quotes. Requests to access these datasets should be directed to Yao Lu, luyao79@sdxiehe.edu.cn.
